# The Inhibitory Mechanisms of Tumor PD-L1 Expression by Natural Bioactive Gallic Acid in Non-Small-Cell Lung Cancer (NSCLC) Cells

**DOI:** 10.3390/cancers12030727

**Published:** 2020-03-19

**Authors:** Dong Young Kang, Nipin Sp, Eun Seong Jo, Alexis Rugamba, Dae Young Hong, Hong Ghi Lee, Ji-Seung Yoo, Qing Liu, Kyoung-Jin Jang, Young Mok Yang

**Affiliations:** 1 Department of Pathology, School of Medicine, Institute of Biomedical Science and Technology, Konkuk University, Seoul 05029, Korea; kdy6459@naver.com (D.Y.K.); nipinsp@gmail.com (N.S.); eses0706@naver.com (E.S.J.); rugambalex@gmail.com (A.R.); 2 Department of Emergency Medicine, School of Medicine, Konkuk University, Seoul 05029, Korea; kuhemhdy@gmail.com; 3 Division of Hematology-Oncology, Department of Internal Medicine, Konkuk University Medical Center, Seoul 05029, Korea; mlee@kuh.ac.kr; 4 Department of Immunology, Hokkaido University Graduate School of Medicine, Sapporo 060-0808, Japan; jiseungy@pop.med.hokudai.ac.jp; 5 Jilin Green Food Engineering Research Institute, Changchun 130000, Jilin, China; liuqing0523@hotmail.com

**Keywords:** natural bioactive compound, gallic acid, EGFR signaling, p53, PD-L1, immunotherapy

## Abstract

Non-small-cell lung cancer (NSCLC) is the most common lung cancer subtype and accounts for more than 80% of all lung cancer cases. Epidermal growth factor receptor (EGFR) phosphorylation by binding growth factors such as EGF activates downstream prooncogenic signaling pathways including KRAS-ERK, JAK-STAT, and PI3K-AKT. These pathways promote the tumor progression of NSCLC by inducing uncontrolled cell cycle, proliferation, migration, and programmed death-ligand 1 (PD-L1) expression. New cytotoxic drugs have facilitated considerable progress in NSCLC treatment, but side effects are still a significant cause of mortality. Gallic acid (3,4,5-trihydroxybenzoic acid; GA) is a phenolic natural compound, isolated from plant derivatives, that has been reported to show anticancer effects. We demonstrated the tumor-suppressive effect of GA, which induced the decrease of PD-L1 expression through binding to EGFR in NSCLC. This binding inhibited the phosphorylation of EGFR, subsequently inducing the inhibition of PI3K and AKT phosphorylation, which triggered the activation of p53. The p53-dependent upregulation of miR-34a induced PD-L1 downregulation. Further, we revealed the combination effect of GA and anti-PD-1 monoclonal antibody in an NSCLC-cell and peripheral blood mononuclear–cell coculture system. We propose a novel therapeutic application of GA for immunotherapy and chemotherapy in NSCLC.

## 1. Introduction

Lung cancer, constituting 18% of all global cancer deaths, is one of the representative causes of death globally [1]. It is classified into two main groups: small-cell lung cancer (SCLC; 15% of all lung cancers) and non-SCLC (NSCLC; 85% of all lung cancers) [2,3]. NSCLC can be further subcategorized into three subtypes: adenocarcinoma, squamous cell carcinoma, and large cell carcinoma [4]. Despite many efforts to treat NSCLC, the overall survival rate is only 15.9% within five years [5]. Further, many patients receiving NSCLC chemotherapies struggle with adverse reactions, drug resistance, and the necessary target specificity of some types of drugs. Thus, unsatisfactory outcomes in NSCLC treatment have motivated researchers to identify novel agents such as natural compounds [6,7,8]. Moreover, the main advantage of such drugs having fewer side effects relative to non-natural drugs has spurred scientists to reveal their molecular mechanisms. 

Gallic acid (3,4,5-trihydroxybenzoic acid; GA), a natural phenolic compound, is widely distributed in natural plants, fruits, and green tea [9,10]. Many studies have reported that GA exhibits anti oxidative, anti mutagenic, anti carcinogenic, antibacterial, antiviral, and anti-inflammatory effects [11,12,13,14,15]. However, the main interest in GA and its derivatives surrounds its anticancer activity. Previous studies have revealed that GA effectively induces selective apoptosis in various cancer cells, including HeLa, HCT-15, SH-SY5Y, and NSCLC cells [16,17,18,19] and inhibits proliferation and migration via regulating fatty acid synthase in TSGH-8301 cells [20]. Recent studies also revealed potential anticancer effects of GA is due to its ability to inhibit cell proliferation and to induce apoptosis *in vivo* [21,22]. 

Programmed cell death ligand-1 (PD-L1), also known as CD274 and B7-H1, is a transmembrane protein expressed on the surface of antigen-presenting cells such as dendritic cells, macrophages, and B-cells. It is also overexpressed and found in various types of cancer [23,24,25,26]. PD-L1 specifically binds to programmed cell death-1 (PD-1), which is an important inhibitory receptor expressed on the surface of immune-related lymphocytes like T-cells, B-cells, and myeloid cells [27]. The binding of PD-L1 to PD-1 inhibits the proliferation, cytokine generation and release, and cytotoxicity of T-cells. Thus, the binding leads to an immunosuppressive effect and allows cancer cells to escape immune eradication via assistance from tumor-specific T-cells [28]. PD-L1 overexpression in cancer cells promotes cancer progression and leads cancer cells to malignancy. Moreover, the intrinsic signal transduction by PD-L1 enhances the survival of cancer cells through increasing the resistance toward proapoptotic stimuli such as interferons [29]. PD-L1 expression at the transcriptional level is regulated individually or cooperatively by many oncogenic transcription factors such as MYC, AP-1, STAT, IRF1, HIF, and NF-κB. Some studies have demonstrated that there is a tendency toward higher PD-L1 expression in *TP53*-mutated and low p53-expression cancer cells, which imply that PD-L1 expression is considerably related to p53 status in cancer cells [30,31,32,33]. In addition, tumor suppressor gene *PTEN*, one of the most frequently mutated genes in human cancers, downregulates PD-L1 expression, which signifies that tumor suppressors play an important role in controlling PD-L1 expression [34,35,36].

Previous research has demonstrated that apigenin, a kind of flavonoid, induces growth-suppressive and proapoptotic effects in melanoma cells. Additionally, such significantly inhibits the expression of interferon (IFN)-γ-induced PD-L1, which may indicate the existence of an immunosuppressive effect shown by a natural compound [37]. In this study, we examined the anticancer and immunosuppressive activities of natural bioactive GA in NSCLC A549 and H292 cells (wild-type p53 and epidermal growth factor receptor (EGFR)), which are kinds of NSCLC cells. The binding of GA to EGFR inhibited the EGFR phosphorylation, leading to the promotion of p53 expression in both A549 and H292 cells. Furthermore, highly expressed p53 decreased PD-L1 protein expression through enhancing the miR-34a related to PD-L1 downregulation at the transcriptional level. As such, our results suggest an immunosuppressive effect of GA toward NSCLC cells, which might imply a potential possibility for clinical application in NSCLC treatment.

## 2. Results

### 2.1. GA Downregulates the PD-L1 Expression in NSCLC Cells

**Figure 1:**
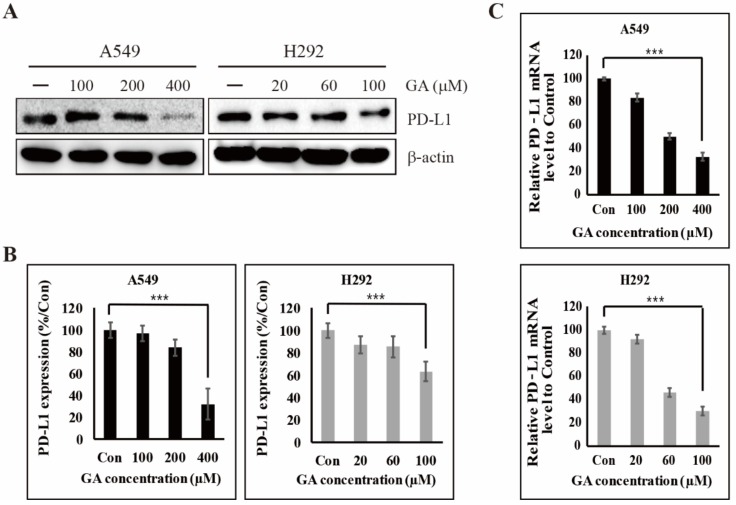
Gallic acid (GA) reduces the programmed death-ligand 1 (PD-L1) expression in non-small-cell lung cancer (NSCLC) cells. (**A**) The expression levels of PD-L1 protein in A549 and H292 cells were detected after GA treatment in concentrations indicated for 48 hours. (**B**) The relative expression levels of PD-L1 protein were determined by densitometry and normalized to β-actin. Data are representative of three independent experiments. *** *p* < 0.001 (*t-*test). (**C**) The expression levels of PD-L1 mRNA in A549 and H292 cells were detected after GA treatment in concentrations indicated for 48 hours. The relative expression levels of PD-L1 mRNA were determined by real time qPCR and normalized to GAPDH mRNA. Data are representative of three independent experiments. *** *p* < 0.001 (*t-*test).

To determine whether GA inhibits the cell proliferation of A549 and H292 cell lines, GA-treated cells were compared with non-treated control cells. The results of MTT assay showed that the cell growth of GA-treated cells is significantly inhibited in a time- and concentration-dependent manner (Figure S1A). This result was confirmed with crystal violet assay by treating GA in A549 and H292 cells for 48 h (Figure S1B). From this data, we respectively identified an IC_50_ dosage of 400 μM in A549 and 100 μM in H292 cell lines at 48 hours, respectively, information which was used for further studies. We checked the same concentration in non-cancerous cells (HUVEC cell line) and found that 400 μM GA inducing around 8% cell death which indicated that this concentration does not make much toxicity in normal cells (Figure S2). Recently, cancer immunotherapy based on PD-1/PD-L1 blockade has shown clinical efficacy in the treatment of multiple cancers [26,28]. In addition, a study of the drug-induced inhibition of PD-L1 expression in cancer cells has been conducted [37]. To investigate whether GA influences PD-L1 expression, we assessed the expression levels of PD-L1 by the impact of GA in NSCLC cells. As shown in Figure 1A, results from western blotting suggested that GA strongly decreases the expression levels of PD-L1 protein in A549 and H292 NSCLC cells. In addition, GA showed a greater than 70% inhibitory effect as compared with in non-treated control cells among A549 cells (Figure 1B). Subsequently, we performed a real time PCR experiment to examine the influence of GA on the messenger RNA (mRNA) expression of PD-L1 in A549 and H292 cell lines. In accordance with data from real time PCR, GA also downregulated the expression of PD-L1 mRNA in a concentration-dependent manner in both A549 and H292 cells (Figure 1C). These results suggest that bioactive natural GA has a significant inhibitory effect on PD-L1 expression (both protein and mRNA) in A549 and H292 cell lines, which imply the potential of using it as an immune anticancer agent.

### 2.2. GA Binds to EGFR and Then Inhibits its Phosphorylation

**Figure 2:**
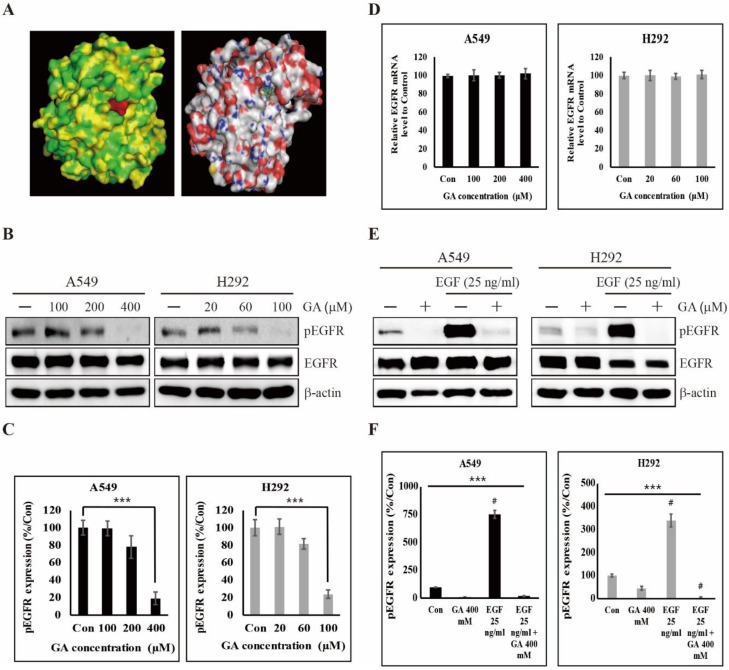
GA binding to epidermal growth factor receptor (EGFR) inhibits the phosphorylation of EGFR. (**A**) Binding of GA (PubChem CID: 370) to the ATP-binding domain of EGFR (Protein Data Bank ID: 4LQM) as determined by molecular docking using AutoDock Vina. (**B**) The expression levels of total EGFR and phosphorylated EGFR (pEGFR) protein in A549 and H292 cells were detected after GA treatment in concentrations indicated for 48 hours. (**C**) The relative levels of pEGFR protein were determined by densitometry and normalized to β-actin. Data are representative of three independent experiments. *** *p* < 0.001 (*t-*test). (**D**) The expression levels of EGFR mRNA in A549 and H292 cells were detected by real time PCR after GA treatment in concentrations indicated for 48 hours. The relative levels of EGFR mRNA were determined and normalized to GAPDH mRNA. Data are representative of three independent experiments. (**E**) A549 and H292 cells for detecting the expression levels of total EGFR and pEGFR protein were treated with or without 25 ng/mL EGF for 15 minutes and then further treated with GA (A549: 400 μM; H292: 100 μM) for 48 hours. (**F**) The relative levels of pEGFR protein were determined by densitometry and normalized to β-actin. Data are representative of three independent experiments. *** *p* < 0.001 (*t-*test). # *p* < 0.001 vs. control.

EGFR phosphorylation induces various oncogenic signaling pathways for cell proliferation, invasion, and metabolic reprogramming in many cancer cells [38]. Therefore, to inhibit EGFR phosphorylation in cancer cells, many clinical applications have been stimulated to develop EGFR tyrosine kinase inhibitors (TKIs) such as erlotinib, gefitinib, and lapatinib [39,40]. In previous studies, we found that the binding of natural compounds to EGFR, causing a decrease in EGFR phosphorylation, inhibited the proliferation, migration, invasion, and angiogenesis of human breast adenocarcinoma cells [41,42]. To understand the impact of GA for EGFR phosphorylation, we identified the binding ability of GA to EGFR. Molecular docking was performed with an AutoDock Vina platform (Oleg Trott, The Scripps Research Institute, La Jolla, CA, USA). We found that GA is docked in the ATP binding site of EGFR, and this result may imply the direct binding of GA to EGFR (Figure 2A). Subsequently, we further performed an immunoblot analysis for understanding whether the GA/EGFR binding influences the phosphorylation of EGFR and found that, GA significantly downregulated the phosphorylation of EGFR in both A549 and H292 cells (EGFR wild-type NSCLC cells) (Figure 2B,C). However, this treatment did not affect the expression levels of total EGFR mRNA (Figure 2D). These results may imply that GA could influence the inhibition of EGFR signal transduction in two NSCLC cells. Moreover, these results led us to investigate the binding specificity of GA to EGFR, where we conducted a competitive binding experiment of GA and EGF (25 ng/mL pre-treatment for 15 min) versus EGFR. Here, GA significantly inhibited EGF-induced EGFR phosphorylation in both A549 and H292 cells (Figure 2E,F). This result suggests that GA binds to EGFR as compared with the natural ligand (EGF) for EGFR, and this act of binding clearly induces the inhibition of EGFR phosphorylation.

### 2.3. GA Reduces the Phosphorylation of PI3K/AKT That is One of the Downstream Targets of EGFR Signaling

**Figure 3:**
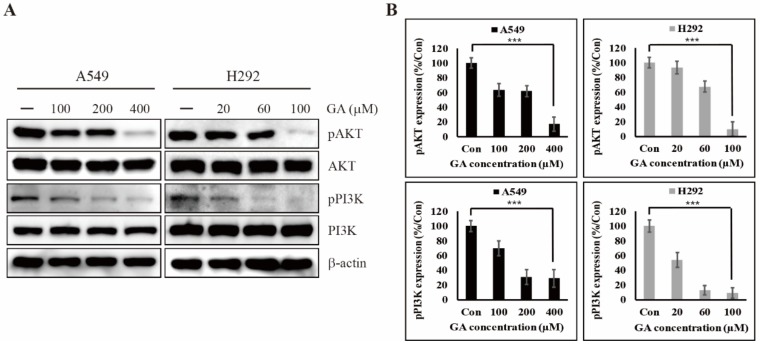
GA inhibits the phosphorylation of AKT and PI3K protein in a GA concentration-dependent manner. (**A**) The expression levels of pAKT and pPI3K protein in A549 and H292 cells were detected after GA treatment in concentrations indicated for 48 hours. (**B**) The relative expression levels of pAKT and pPI3K protein were determined by densitometry and normalized to β-actin. Data are representative of three independent experiments. *** *p* < 0.001 (*t-*test).

EGF/EGFR signal transduction has been known to lead to the constitutive activation of downstream signaling pathways associated with MAPKs, STAT3, and PI3K for regulating PD-L1 expression in various cancer cells [43]. A previous study found that the PD-L1 expression of EGFR–mutated PC-9 cells was significantly higher than those of EGFR wild-type LU-99, A549, and PC-14 cells. In EGFR inhibitor experiments, the EGFR TKI gefitinib induced a lower expression of phosphorylated AKT and STAT3, which prompted the downregulation of PD-L1 expression [44]. To determine a key EGFR-downstream pathway associated with PD-L1 expression, we used an immunoblot analysis. As shown in Figure S3, GA treatment did not inhibit the phosphorylation of JAK2/STAT3, which is one of the main pathways. However, GA efficiently controlled the PI3K/AKT pathway by inhibiting their phosphorylation but not total protein level (Figure 3A,B). These results clearly show that the regulation of PI3K/AKT phosphorylation by GA could be responsible for PD-L1 expression in both A549 and H292 cells. Moreover, the downregulation of PI3K/AKT phosphorylation by GA may indicate a beneficial effect in terms of controlling various oncogenic signals, such as cellular proliferation, invasion, angiogenesis, and metastasis.

### 2.4. GA Activates the Expression of Tumor Suppressor Factor p53 for Inhibiting the Expression of PD-L1

**Figure 4:**
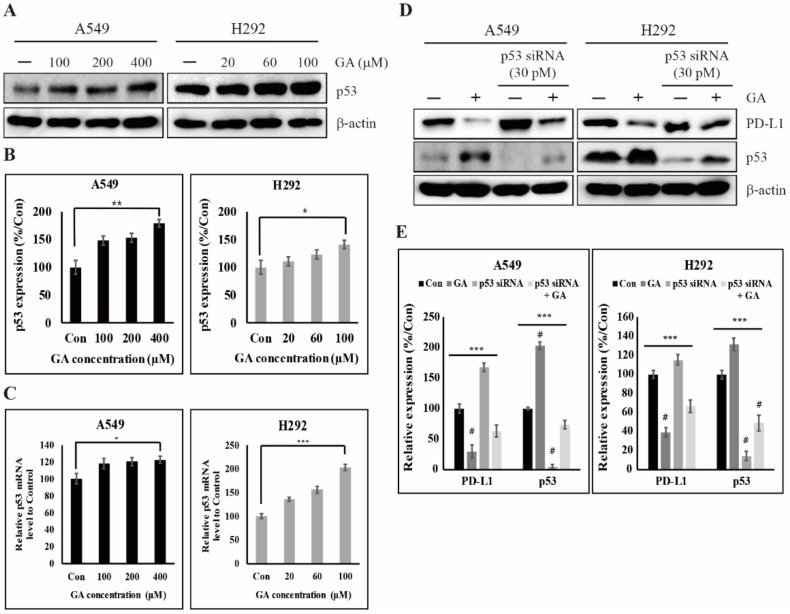
GA increased p53 expression. (**A**) The expression levels of p53 protein in A549 and H292 cells were detected after GA treatment in concentrations indicated for 48 hours. (**B**) The relative expression levels of p53 protein were determined by densitometry and normalized to β-actin. Data are representative of three independent experiments. * *p* < 0.05 and ** *p* < 0.01 (*t-*test). (**C**) The expression levels of p53 mRNA in A549 and H292 cells were detected by real time qPCR after GA treatment in concentrations indicated for 48 hours. The relative expression levels of p53 mRNA were determined and normalized to GAPDH mRNA. Data are representative of three independent experiments. * *p* < 0.05 and *** *p* < 0.001 (*t-*test). (**D**) The expression levels of p53 and PD-L1 protein in A549 and H292 cells, treated with GA (A549: 400 μM; H292: 100 μM) or 30 pM p53 siRNA, were detected by western blotting at 48 hours. (**E**) The relative expression levels of p53 and PD-L1 protein were determined using densitometry and normalized to β-actin. Data are representative of three independent experiments. *** *p* < 0.001 (ANOVA); # *p* < 0.001 vs. control.

The tumor suppressor factor p53 plays an important role in cell-cycle arrest and apoptosis induction in response to oncogenic or other stresses for the prevention of cancer development. However, it is downregulated or mutated in an inactive form in almost all human cancer cells. A previous study found that p53 is led into Mdm2-mediated ubiquitination and degradation by PI3K/AKT signal transduction in breast cancer MCF-7 cells but not p53 mRNA [45]. Furthermore, p53-regulated IFN-γ induced PD-L1 expression in melanoma cells [32]. To investigate the effect on p53 by GA, we checked the protein levels of p53 with or without GA treatment in A549 and H292 cells and found that GA upregulates the expression levels of p53 protein in a concentration-dependent manner (Figure 4A,B). In addition, the expression levels of p53 protein were nearly doubled in A549 cells. Further, the mRNA levels of p53 identified by real time PCR showed a significant increase in a GA concentration-dependent manner in H292 cells as well as in A549 cells (Figure 4C). From these data, although a previous study revealed that PI3K/AKT signaling induced by their phosphorylation regulates only p53 protein levels, the PI3K/AKT signaling controlled by GA plays a key role in regulating both protein and mRNA levels of p53. These results additional imply that GA regulates p53 from mRNA levels through the downregulation of PI3K/AKT phosphorylation. To further understand the role of p53 in PD-L1 regulation, we used GA with or without p53 siRNA and determined whether specific gene silencing influences PD-L1 expression in A549 and H292 cells. As shown in Figure 4D,E, the gene silencing of p53 significantly affected the increase in PD-L1 proteins compared to non-treated control, which was decreased by GA treatment. In contrast, the effect by GA regulated the protein levels of p53 and PD-L1 in two NSCLC cells. These results imply that the regulation of PD-L1 by GA is indirectly controlled by way of inducing an increase in p53 protein level. In addition, the upregulation of p53 by GA may induce various p53-mediated anti-oncogenic factors such as the regulation of miRNA.

### 2.5. GA Upregulates p53-Dependent MiR-34a for Inhibiting the Expression of PD-L1

**Figure 5:**
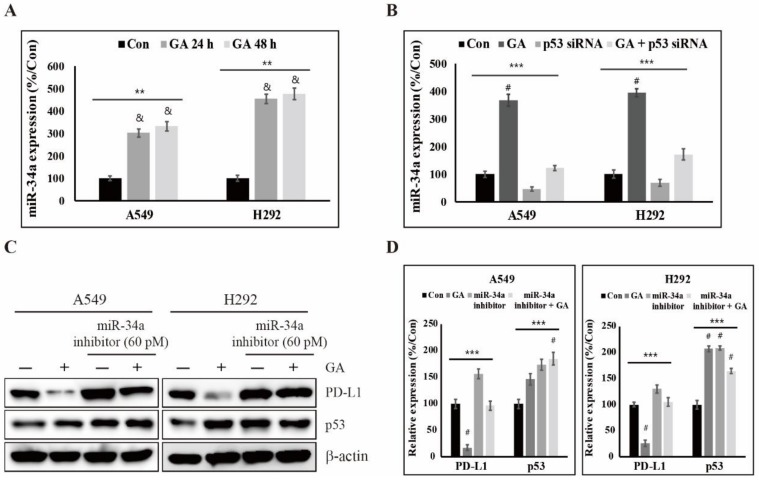
GA upregulates the PD-L1 expression by p53 via miR-34a. (**A**) Relative expression levels of miR34a after treatment of GA (A549: 400 μM; H292: 100 μM) for 24 and 48 hours. Data are representative of three independent experiments. ** *p* < 0.01 (ANOVA); & *p* < 0.01 vs. control. (**B**) The expression levels of miR-34a in A549 and H292 cells, treated with GA (A549: 400 μM; H292: 100 μM), p53 siRNA (60 pM) or GA plus p53 siRNA were detected by RT-PCR at 48 hours. The relative expression levels of miR-34a were determined using densitometry and normalized to U6. Data are representative of three independent experiments. *** *p* < 0.001 (ANOVA); # *p* < 0.001 vs. control. (**C**) The expression levels of p53 and PD-L1 protein in A549 and H292 cells, treated with GA (A549: 400 μM; H292: 100 μM) or 30 pM miR-34a inhibitor, were detected by western blotting at 48 hours. (**D**) The relative expression levels of p53 and PD-L1 protein were determined by densitometry and normalized to β-actin. Data are representative of three independent experiments. *** *p* < 0.001 (ANOVA); # *p* < 0.001 vs. control.

miRNAs, a family of small noncoding RNAs, regulate wide biological processes including carcinogenesis, which severely is dysregulated in many cancer cells. Some miRNAs such as miR-513 and miR-570 directly target PD-L1 [46,47]. However, p53 indirectly regulates the expression levels of PD-L1 through inducing miR-34a in cancer cells [33]. Although many studies have shown results for the regulation of PD-L1 expression directly by miRNA, detailed studies of the actions brought on indirectly by p53 via drugs including natural compounds is poorly understood. To understand the expression level of miR-34a by GA, we performed a real time PCR experiment because miR-34a is a well-known molecule transcriptionally induced by p53. As shown in Figure 5A, we found that it was significantly increased in a time- and GA concentration-dependent manner in both A549 and H292 cells. To further investigate miR-34a regulation by GA via p53, we additionally used p53 siRNA. The expression levels of miR-34a were decreased by p53 siRNA, but their expression levels were slightly increased by additional GA (Figure 5B). These results clearly suggest that miR-34a expression is regulated by GA-dependent p53. Additionally, we used a miR-34a inhibitor with or without GA to determine a more detailed interrelation analysis in the regulation of PD-L1 expression. In this experiment, we demonstrated that the inhibition of miR-34a function by its inhibitor is induced into an increase of PD-L1 protein which reversed by GA, but not p53 (Figure 5C,D). These results support that the expression of PD-L1 is regulated via miR-34a-induction through GA-dependent p53 in A549 and H292 cells.

### 2.6. The Downregulation of PD-L1 Expression by GA Induces the Combination Effect with PD-1 Blockade

**Figure 6:**
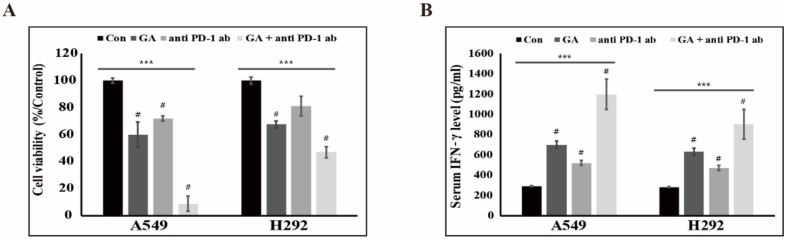
GA enhances the combination effect with anti-PD-1 mAb in an NSCLC-cell and peripheral blood mononuclear-cell (PBMC) coculture system. (**A**) The cell survival rates of NSCLC cells cocultured with PBMCs were examined after treatment with mock, GA (A549: 200 μM; H292: 50 μM), anti-PD-1 mAb (5 μg/mL), or both GA and anti-PD-1 mAb for 48 hours. *** *p* < 0.001 (ANOVA); # *p* < 0.001 vs. control. (**B**) The levels of interferon (IFN)-γ from the supernatants of the coculture system were measured by ELISA also following treatment with mock, GA (A549: 200 μM; H292: 50 μM), anti-PD-1 mAb (5 μg/mL), or both GA and anti-PD-1 mAb for 48 hours. *** *p* < 0.001 (ANOVA); # *p* < 0.001 vs. control.

**Figure 7:**
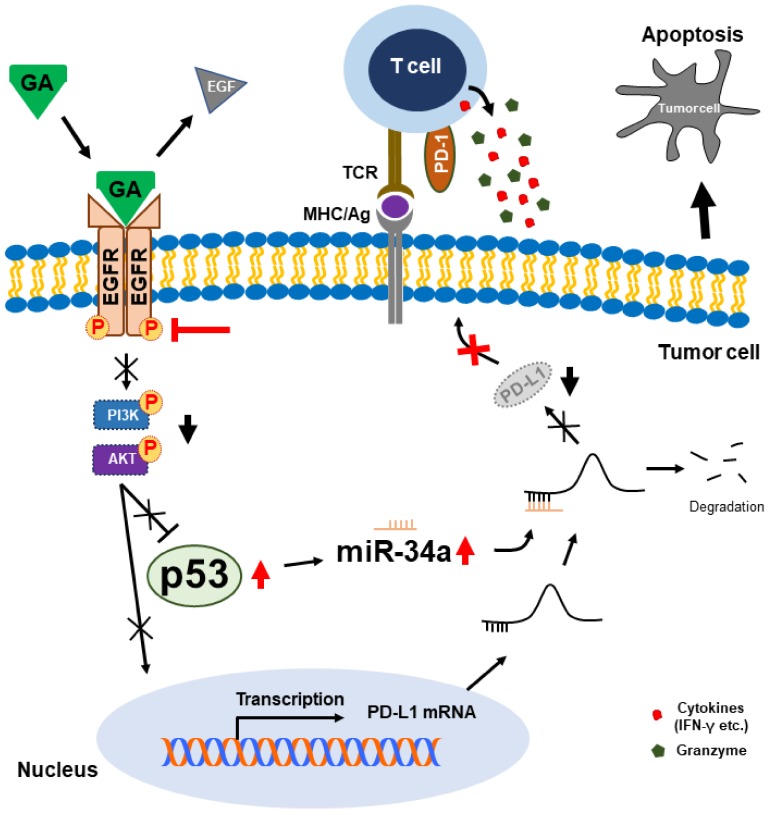
Molecular regulatory mechanism of programmed death-ligand 1 (PD-L1) by natural bioactive gallic acid in NSCLC cells and proposed combination effect for NSCLC immunotherapy.

To test the combination effect of PD-1 blockade and GA on antitumor activity, we evaluated cytotoxicity in an NSCLC-cell and peripheral blood mononuclear-cell (PBMC) coculture system in the presence of the anti-PD-1 monoclonal antibody (mAb) nivolumab, GA, or both. We observed a considerable apoptotic effect in the presence of both PD-1 mAb and GA in A549 and H292 cells (Figure 6A). Further, GA reduced the viability of cancer cells more effectively in comparison with a single blockade of PD-1 with PD-1 mAb. These results may indicate that the decrease of PD-L1 expression by GA regulates not only reducing survival signals of PD-L1 downstream but also activates the T-cell-mediated immune response. To further investigate the combination effect on PBMC cytokine expression, we performed an IFN-γ analysis by enzyme-linked immunosorbent assay (ELISA). As shown in Figure 6B, GA treatment was observed to slightly increase the IFN-γ level more so than a single blockade of PD-1 with PD-1 mAb. In addition, treatment with both GA and PD-1 mAb considerably enhanced the IFN-γ production in the supernatant of the NSCLC-cell and PBMC coculture system. These results suggest that the decrease in PD-L1 expression brought about by GA enhances the effect observed with PD-1 mAb in the production of IFN-γ. Figure 7 is a graphical abstract which gave the conclusion of all these results. We checked the effect of this combination in a non-cancerous cell (HUVEC cell line) and found that these combination does not induce much cell toxicity in non-cancerous cells (Figure S3). 

## 3. Discussion

An important concept in cancer treatment is that the cancerous cells should ideally be removed without influencing normal cells. Chemotherapy is the most common type of treatment, where chemicals or drugs to destroy cancer cells and cancer microenvironments are applied. Genomic studies such as those on *TP53, BCL2,* and *c-MYC* have accelerated the effective application of chemotherapy for developing anticancer drugs and reagents in cancer treatment [48,49,50]. Anticancer drugs, according to their mechanisms of action, are generally classified as either alkylating agents for damaging cancer cell DNA, antimetabolites for replacing the normal building blocks of RNA and DNA, or antibiotics for interfering with the enzymes involved in DNA replication [51,52,53]. 

Although observed therapeutic issues for NSCLC are still deemed to be unsatisfactory because of multidrug resistance and adverse effects [54,55], chemical drugs such as vinorelbine and cisplatin have been tested in NSCLC treatment [56,57]. To overcome these problems, the combined effects of two chemotherapy drugs including cisplatin or carboplatin plus one other drug have often been deployed to treat early-stage NSCLC. Despite many efforts, these chemotherapy-based regimens seem to have reached a therapeutic limit. Recently, many studies have reported the potential possibility of applying natural compounds in the treatment or control of various cancerous diseases. In previous studies, we demonstrated various anticancer effects of natural compounds [41,58,59]. Moreover, combination treatment using a chemotherapy drug and naturally derived drugs showed more effective anticancer effects, which imply that such might reduce the burden of adverse effects brought on by chemotherapy drugs alone [60]. However, a therapeutic strategy using natural compounds is difficult to apply without knowing the specific targets, which is one of the disadvantages of use. Thus, a targeted study focused on using natural compounds is essential to achieve more effective anticancer treatment. Many studies have investigated a phenolic natural compound, gallic acid, that acts as an anticancer agent against various cancers [16,17,19,20]. Nevertheless, these studies did not identify where the target position of GA is against various cancer cells or did they reveal detailed molecular mechanisms underlying the anticancer effects of GA in cancer cell death. In this study, we demonstrated that GA influences cancer cell viability and specifically binds to the tyrosine kinase receptor, EGFR in NSCLC cell lines.

EGFR is a cell-surface protein that binds with epidermal growth factor (EGF) [61]. EGFR often is mutated and/or overexpressed in several types of human cancers, including lung, ovary, breast, head, and neck cancer, and it serves to modulate the growth, differentiation, signaling, adhesion, migration, and survival of cancer cells. Usually, EGF-mediated EGFR phosphorylation induces three main signal transductions including JAK-STAT, KRAS-ERK, and PI3K-AKT-mTOR. These pathways are known to be involved in the growth, proliferation, inhibition of apoptosis, angiogenesis, and invasion of cancer cells [62,63]. For this reason, EGFR has been regarded as an attractive candidate for anticancer treatment because of its multifunctional role in tumorigenesis [38]. To date, two monoclonal antibodies, cetuximab and panitumumab, capable of inhibiting EGF or growth factor-mediated signaling pathways have been used for cancer therapy [64]. In addition, several TKIs such as erlotinib and gefitinib have been employed for the inhibition of EGFR phosphorylation. In this study, we demonstrated that GA inhibits EGFR phosphorylation by binding to EGFR in two NSCLC cells. Moreover, GA showed binding specificity and inhibited EGFR phosphorylation despite EGF-binding. These results may imply that GA is a selective and potent inhibitor against EGFR phosphorylation. Furthermore, the inhibition of EGFR phosphorylation by GA induced the downregulation of phosphorylated PI3K and AKT. Previous studies revealed that the inhibition of EGFR TKI-mediated EGFR phosphorylation induces the downregulation of phospho-PI3K and AKT [65,66]. Therefore, GA, which showed a similar effect to that of TKIs, may be a useful drug candidate for NSCLC treatment. 

The tumor suppressor p53 is a transcription factor and plays a pivotal role in cell-cycle, DNA repair, senescence, and apoptosis [67,68,69]. Under various stresses such as DNA damage, p53 is phosphorylated and acetylated via posttranslational modification and then it is translocated to the nucleus for trans-activating numerous target genes that direct processes including cell-cycle arrest and/or apoptosis. Mutations of *TP53* have been discovered in more than 50% of human cancers and p*53* mutation leads to not only the loss of cancer suppressive functions but also the acquisition of additional oncogenic functions such as growth and survival [70]. Wild-type p53 proteins (WTp53) are frequently downregulated because of their function of tumor suppression in many cancer cells. Previous studies have identified that the downregulation of WTp53 is associated with EGFR signal-mediated PI3K/AKT pathway activation in cancer cells [65,66,71,72]. As mentioned above, we demonstrated that the downregulation of EGFR phosphorylation by GA leads to the inhibition of PI3K and AKT phosphorylation. The decrease in their phosphorylation by GA induced the upregulation of WTp53 protein and mRNA in A549 and H292 cells. Furthermore, the competitive activity of GA in an EGF-dependent condition suggested that binding of GA to EGFR, may associated with the upregulation of p53 through inhibiting EGFR/PI3K/AKT4 phosphorylation. These results also suggest that natural bioactive GA may have a potential role as a chemotherapeutic drug for NSCLC treatment. Although many studies have revealed that p53 is related to some immune responses including IFN signaling [73,74], the expression of inflammatory cytokines and Toll-like receptors [75,76,77], and the activation of T- and natural killer cells [78], the correlation of p53 and tumor immune evasion is poorly understood. Recently, some studies reported that p53 interacts with the apoptotic pathway by regulating miRNAs in cancer cells [79,80]. Furthermore, the effect on p53 was augmented in miR-34a expression, which leads to decreased expression levels of PD-L1 in NSCLC cells [33]. Interestingly, we found that bioactive GA decreased the protein and mRNA levels of PD-L1 as compared with the control experiment, and the expression levels of p53 and miR-34a were upregulated by GA in NSCLC cells. These results propose that GA controls the expression of PD-L1 by regulating the p53–miR-34a pathway. 

Finally, studies have revealed that PD-L1 expression in cancer cells enhances cell proliferation and resistance toward pro-apoptotic stimuli [29,81]. Furthermore, PD-L1 expression in cancer also enhances PD-L1-mediated tumor immune resistance from cytotoxic cluster of differentiation (CD)8 T-cells through the PD-1/PD-L1 blockade [82]. Thus, inhibition of PD-L1 expression will activate cytotoxic CD8 T-cell responses to various cancers. This approach has been labeled as PD-1/PD-L1 based-immunotherapy. Recently, many clinical approaches and successes are emerging through PD-1/PD-L1 blockade therapy. As mentioned above, we found that GA decreases PD-L1 expression in A549 and H292 cells. In combination experiments with a human monoclonal anti-PD-1 mAb (nivolumab), GA exhibited a more effective effect regarding cancer cell viability. In accordance with the decreased expression levels of PD-L1 by GA, the experimental condition involving anti-PD-1 mAb decreased NSCLC cell viability and oppositely increased the level of IFN-γ in the NSCLC-cell and PBMC coculture system. 

## 4. Materials and Methods 

### 4.1. Cell Lines and Cell Culture

Roswell Park Memorial Institute (RPMI) 1640 media and a penicillin–streptomycin solution was purchased from Gibco (Gaithersburg, MD, USA). Fetal bovine serum (FBS) was purchased from Sigma-Aldrich (St. Louis, MO, USA). Trypsin ethylenediaminetetraacetic acid (0.05%) was obtained from Gibco (Gaithersburg, MD, USA). The human NSCLC cell lines H292 (no. 21848; Korean Cell Line Bank, Seoul, South Korea) and A549 (CCL-185; American Type Culture Collection, Manassas, VA, USA) were cultured in RPMI-1640 supplemented with 10% FBS and antibiotics (1% penicillin–streptomycin) at 37 °C with 5% CO_2_. For each experiment, at 70% to 80% confluence, cells were gently washed twice with phosphate-buffered saline. Unless otherwise specified, cells were treated with 100 μM of GA (in H292 cells) or 400 μM of GA (in A549 cells) for 48 hours at 37°C under an atmosphere of 5% CO_2_. 

### 4.2. Immunoblotting

Whole-cell lysates were prepared by radioimmunoprecipitation assay buffer (EMD Millipore, Burlington, MA, USA) containing phosphatase and protease inhibitors. Antibodies specific for β-actin (sc-47778), p53 (sc-126) and secondary antibodies (antimouse (sc-516102), and antirabbit (sc-2357) antibody) were obtained from Santa Cruz Biotechnology, Inc. (Dallas, TX, USA). pEGFR (#2234), EGFR (#3776s), pAKT (#4060), AKT (#4691), pPI3K (#4228), and PI3K (#4257) antibodies were purchased from Cell Signaling Technology Inc. (Danvers, MA, USA). PD-L1 (R30949) antibody was purchased from NSJ Bioreagents (San Diego, CA, USA). Recombinant human EGF (AF-100-15) was purchased from PeproTech Inc. (Rocky Hill, NJ, USA). 

### 4.3. Real Time Quantitative PCR (qPCR)

Total RNA was isolated with the RNeasy Mini Kit (Qiagen GmbH, Hilden, Germany) according to the manufacturer’s protocol. The extracted RNA was quantified spectrophotometrically at 260 nm, and cDNA was synthesized at 42 °C for 1 h and 95 °C for 5 min with a first-strand cDNA synthesis kit (K-2041; Bioneer Corporation, Daejeon, Korea) and oligo d(T) primers. Real-time qPCR was conducted in a thermal cycler (C1000 Thermal Cycler, Bio-Rad, Hercules, CA) as follows: 2 μL diluted cDNA was added to diluted forward and reverse primers (1 μL each, 100 pM) and 10 μL TB Green Advantage Premix (Takara Bio, Japan) according to the manufacturer’s instructions. We used the following primers for EGFR, sense 5’- TGGCAGTGTCTTAGCTGGTTGT -3’ and anti-sense 5’- TGCACTCAGAGAGCTCAGGA -3’, for PD-L1, sense 5’- TGCCAGGCATTGAATCTACA -3’ and anti-sense 5’- GGCCTATTTCCTCCTCTTGG -3’, for p53, sense 5’- AGGCCTTGGAACTCAAGGAT -3’ and anti-sense 5’- CAGTCTGAGTCAGGCCCTTC -3’, and for miR34a analysis: for miR34a, sense 5’- TGGCAGTGTCTTAGCTGGTTGT -3’. The measurement was carried out in triplicate. The relative expression of target genes was normalized to GAPDH or U6 snRNA. 

### 4.4. Transfections of siRNA and miRNA

Lung cancer cells (1 × 10^5^ cells) were seeded in six-well plates and grown to 60% confluence. The cells were then transfected with p53 siRNA (sc-29435; Santa Cruz Biotechnology, Dallas, TX, USA) or miR-34a inhibitor (AM 17000; Thermo Fisher Scientific, Inc., Waltham, MA, USA) using Lipofectamine transfection reagent (Thermo Fisher Scientific, Inc., Waltham, MA, USA). After 48 hours, transfected cells additionally were cultured with/without GA for an additional 48 hours under a cell culture condition. 

### 4.5. NSCLC-Cell and PBMC Co-Culture Experiments

Lung cancer cells (5 × 10^4^ cells) were seeded in 24-well plate until 70% to 80% confluence under a cell culture condition. Human PBMCs were isolated by Ficoll Paque density centrifugation from peripheral blood donated by healthy volunteers using Lymphoprep™ and SepMate™-50 (Stemcell Technologies, Vancouver, Canada). Then, the acquired PBMCs were added into each coculture system at a PBMCs/attached NSCLC cells ratio of 5:1. Some cocultured wells were treated with 5 μg/mL of anti-PD-1 mAb (nivolumab, #A1307; BioVision, Milpitas, CA, USA) and/or GA (A549: 200 μM and H292: 50 μM) and cultured for 48 hours. After 48 hours of co-culture, the culture supernatant was used to analyze the human IFN-γ level, while the viability of attached NSCLC cells was analyzed by MTT assay. The human IFN-γ level in cell-free supernatant was determined using an ELISA kit (#430104; BioLegend, San Diego, CA, USA) according to the manufacturer’s protocol.

### 4.6. Statistical Analyses 

All experiments were performed at least three times. Results are expressed as means ± standard errors of the mean. Statistical analyses were conducted using a one-way analysis of variance (ANOVA) or the Student’s t-test. The one-way ANOVA was performed with Duncan’s multiple-range test as a post-hoc test. Analyses were performed using the SAS 9.3 program (SAS Institute, Inc., Cary, NC, USA). A *p*-value of less than 0.05 was taken to indicate a statistically significant difference.

## 5. Conclusions

In summary, our results constitute the first study to disclose the detailed mechanism of PD-L1 downregulation, which could be mediated by bioactive natural GA in NSCLC cells. Moreover, we demonstrated that GA might not only directly inhibit cancer cell survival through the upregulation of tumor suppressor p53 but also indirectly enhance antitumor immunity through the downregulation of PD-L1. Thus, our findings additionally pave the way for further research on bioactive natural compounds to study its efficiency in combinations with immune checkpoint-based therapies and chemotherapeutic agents.

## Supplementary Materials

The following are available online at https://www.mdpi.com/2072-6694/12/3/727/s1, Figure S1: Effects of GA on A549 and H292 cell viability. (A) MTT-based evaluation of A549 and H292 cell viability in different time- and GA concentration-dependent manners. Data are representative of three independent experiments. *** *p* < 0.001 (*t-*test). (B) A549 and H292 cell lines were treated with increasing concentration of GA for 48 h and the viability was checked using the crystal violet method. Data are representative of three independent experiments. *** *p* < 0.001 (*t-*test). Figure S2: Effects of GA on Huvec cell viability. The cell survival rates of Huvec cells were examined after treatment with 400 μM GA, anti-PD-1 mAb (5 μg/mL), or both GA and anti-PD-1 mAb for 48 hours. Figure S3: Effect of GA in Jak2/STAT3 pathway. (A) The expression levels of pSTAT3 and pJAK2 protein in A549 and H292 cells were detected after GA treatment in concentrations indicated for 48 h. Data are representative of three independent experiments. * *p* < 0.05 (*t-*test). (B) Relative protein levels pJak2 and pSTAT3 were determined by densitometry analysis and normalized to β-actin. Data are representative of three independent experiments.

## References

[1] Reck M., Heigener D.F., Mok T., Soria J.C., Rabe K.F. (2013). Management of non-small-cell lung cancer: Recent developments. Lancet.

[2] Bender E. (2014). Epidemiology: The dominant malignancy. Nature.

[3] Inamura K. (2017). Lung Cancer: Understanding its molecular pathology and the 2015 WHO classification. Front. Oncol..

[4] Brambilla E., Travis W.D., Colby T.V., Corrin B., Shimosato Y. (2001). The new World Health Organization classification of lung tumours. Eur. Respir. J..

[5] Ettinger D.S., Akerley W., Borghaei H., Chang A.C., Cheney R.T., Chirieac L.R., D’Amico T.A., Demmy T.L., Govindan R., Grannis F.W. (2013). Non-Small Cell Lung Cancer, Version 2.2013 Featured Updates to the NCCN Guidelines. J. Natl. Compr. Cancer Netw..

[6] Di S.Y., Fan C.X., Yang Y., Jiang S., Liang M.M., Wu G.L., Wang B.D., Xin Z.L., Hu W., Zhu Y.F. (2015). Activation of endoplasmic reticulum stress is involved in the activity of icariin against human lung adenocarcinoma cells. Apoptosis.

[7] Ma Z.Q., Yang Y., Fan C.X., Han J., Wang D.J., Di S.Y., Hu W., Liu D., Li X.F., Reiter R.J. (2016). Melatonin as a potential anticarcinogen for non-small-cell lung cancer. Oncotarget.

[8] Aung T.N., Qu Z.P., Kortschak R.D., Adelson D.L. (2017). Understanding the Effectiveness of Natural Compound Mixtures in Cancer through Their Molecular Mode of Action. Int. J. Mol. Sci..

[9] Shahrzad S., Aoyagi K., Winter A., Koyama A., Bitsch I. (2001). Pharmacokinetics of gallic acid and its relative bioavailability from tea in healthy humans. J. Nutr..

[10] Abdelwahed A., Bouhlel I., Skandrani I., Valenti K., Kadri M., Guiraud P., Steiman R., Mariotte A.M., Ghedira K., Laporte F. (2007). Study of antimutagenic and antioxidant activities of gallic acid and 1,2,3,4,6-pentagalloylglucose from Pistacia lentiscus. Confirmation by microarray expression profiling. Chem. Biol. Interact..

[11] Velderrain-Rodriguez G.R., Torres-Moreno H., Villegas-Ochoa M.A., Ayala-Zavala J.F., Robles-Zepeda R.E., Wall-Medrano A., Gonzalez-Aguilar G.A. (2018). Gallic Acid Content and an Antioxidant Mechanism Are Responsible for the Antiproliferative Activity of ‘Ataulfo’ Mango Peel on LS180 Cells. Molecules.

[12] Kim S.W., Han Y.W., Lee S.T., Jeong H.J., Kim S.H., Kim I.H., Lee S.O., Kim D.G., Kim S.Z., Park W.H. (2008). A superoxide anion generator, pyrogallol, inhibits the growth of HeLa cells via cell cycle arrest and apoptosis. Mol. Carcinog..

[13] Sorrentino E., Succi M., Tipaldi L., Pannella G., Maiuro L., Sturchio M., Coppola R., Tremonte P. (2018). Antimicrobial activity of gallic acid against food-related Pseudomonas strains and its use as biocontrol tool to improve the shelf life of fresh black truffles. Int. J. Food Microbiol..

[14] Lee J.H., Oh M., Seok J.H., Kim S., Lee D.B., Bae G., Bae H.I., Bae S.Y., Hong Y.M., Kwon S.O. (2016). Antiviral Effects of Black Raspberry (Rubus coreanus) Seed and Its Gallic Acid against Influenza Virus Infection. Viruses-Basel.

[15] Dludla P.V., Nkambule B.B., Jack B., Mkandla Z., Mutize T., Silvestri S., Orlando P., Tiano L., Louw J., Mazibuko-Mbeje S.E. (2019). Inflammation and Oxidative Stress in an Obese State and the Protective Effects of Gallic Acid. Nutrients.

[16] You B.R., Moon H.J., Han Y.H., Park W.H. (2010). Gallic acid inhibits the growth of HeLa cervical cancer cells via apoptosis and/or necrosis. Food Chem. Toxicol..

[17] Subramanian A.P., Jaganathan S.K., Mandal M., Supriyanto E., Muhamad I.I. (2016). Gallic acid induced apoptotic events in HCT-15 colon cancer cells. World J. Gastroentero..

[18] Tang H.M., Cheung P.C.K. (2019). Gallic Acid Triggers Iron-Dependent Cell Death with Apoptotic, Ferroptotic, and Necroptotic Features. Toxins.

[19] Phan A.N., Hua T.N., Kim M.K., Vo V.T., Choi J.W., Kim H.W., Rho J.K., Kim K.W., Jeong Y. (2016). Gallic acid inhibition of Src-Stat3 signaling overcomes acquired resistance to EGF receptor tyrosine kinase inhibitors in advanced non-small cell lung cancer. Oncotarget.

[20] Liao C.C., Chen S.C., Huang H.P., Wang C.J. (2018). Gallic acid inhibits bladder cancer cell proliferation and migration via regulating fatty acid synthase (FAS). J. Food Drug Anal..

[21] Bhattacharya S., Muhammad N., Steele R., Peng G., Ray R.B. (2016). Immunomodulatory role of bitter melon extract in inhibition of head and neck squamous cell carcinoma growth. Oncotarget.

[22] Raina K., Rajamanickam S., Deep G., Singh M., Agarwal R., Agarwal C. (2008). Chemopreventive effects of oral gallic acid feeding on tumor growth and progression in TRAMP mice. Mol. Cancer Ther..

[23] Baitsch L., Baumgaertner P., Devevre E., Raghav S.K., Legat A., Barba L., Wieckowski S., Bouzourene H., Deplancke B., Romero P. (2011). Exhaustion of tumor-specific CD8(+) T cells in metastases from melanoma patients. J. Clin. Investig..

[24] Pauken K.E., Wherry E.J. (2015). Overcoming T cell exhaustion in infection and cancer. Trends Immunol..

[25] Sharma P., Allison J.P. (2015). The future of immune checkpoint therapy. Science.

[26] Zou W., Wolchok J.D., Chen L. (2016). PD-L1 (B7-H1) and PD-1 pathway blockade for cancer therapy: Mechanisms, response biomarkers, and combinations. Sci. Transl Med..

[27] Havel J.J., Chowell D., Chan T.A. (2019). The evolving landscape of biomarkers for checkpoint inhibitor immunotherapy. Nat. Rev. Cancer.

[28] Swaika A., Hammond W.A., Joseph R.W. (2015). Current state of anti-PD-L1 and anti-PD-1 agents in cancer therapy. Mol. Immunol..

[29] Escors D., Gato-Canas M., Zuazo M., Arasanz H., Garcia-Granda M.J., Vera R., Kochan G. (2018). The intracellular signalosome of PD-L1 in cancer cells. Signal Transduct. Target. Ther..

[30] Pascual M., Mena-Varas M., Robles E.F., Garcia-Barchino M.J., Panizo C., Hervas-Stubbs S., Alignani D., Sagardoy A., Martinez-Ferrandis J.I., Bunting K.L. (2019). PD-1/PD-L1 immune checkpoint and p53 loss facilitate tumor progression in activated B-cell diffuse large B-cell lymphomas. Blood.

[31] Cha Y.J., Kim H.R., Lee C.Y., Cho B.C., Shim H.S. (2016). Clinicopathological and prognostic significance of programmed cell death ligand-1 expression in lung adenocarcinoma and its relationship with p53 status. Lung Cancer.

[32] Thiem A., Hesbacher S., Kneitz H., di Primio T., Heppt M.V., Hermanns H.M., Goebeler M., Meierjohann S., Houben R., Schrama D. (2019). IFN-gamma-induced PD-L1 expression in melanoma depends on p53 expression. J. Exp. Clin. Cancer Res..

[33] Cortez M.A., Ivan C., Valdecanas D., Wang X., Peltier H.J., Ye Y., Araujo L., Carbone D.P., Shilo K., Giri D.K. (2016). PDL1 Regulation by p53 via miR-34. J. Natl. Cancer Inst..

[34] Wang X., Cao X., Sun R., Tang C., Tzankov A., Zhang J., Manyam G.C., Xiao M., Miao Y., Jabbar K. (2018). Clinical Significance of PTEN Deletion, Mutation, and Loss of PTEN Expression in De Novo Diffuse Large B-Cell Lymphoma. Neoplasia.

[35] Xu C., Fillmore C.M., Koyama S., Wu H., Zhao Y., Chen Z., Herter-Sprie G.S., Akbay E.A., Tchaicha J.H., Altabef A. (2014). Loss of Lkb1 and Pten leads to lung squamous cell carcinoma with elevated PD-L1 expression. Cancer cell.

[36] Buchakjian M.R., Merritt N.M., Moose D.L., Dupuy A.J., Tanas M.R., Henry M.D. (2017). A Trp53fl/flPtenfl/fl mouse model of undifferentiated pleomorphic sarcoma mediated by adeno-Cre injection and in vivo bioluminescence imaging. PLoS ONE.

[37] Xu L., Zhang Y., Tian K., Chen X., Zhang R., Mu X., Wu Y., Wang D., Wang S., Liu F. (2018). Apigenin suppresses PD-L1 expression in melanoma and host dendritic cells to elicit synergistic therapeutic effects. J. Exp. Clin. Cancer Res..

[38] Sigismund S., Avanzato D., Lanzetti L. (2018). Emerging functions of the EGFR in cancer. Mol. Oncol..

[39] Bell D.W., Lynch T.J., Haserlat S.M., Harris P.L., Okimoto R.A., Brannigan B.W., Sgroi D.C., Muir B., Riemenschneider M.J., Iacona R.B. (2005). Epidermal growth factor receptor mutations and gene amplification in non-small-cell lung cancer: Molecular analysis of the IDEAL/INTACT gefitinib trials. J. Clin. Oncol..

[40] Erlichman C., Hidalgo M., Boni J.P., Martins P., Quinn S.E., Zacharchuk C., Amorusi P., Adjei A.A., Rowinsky E.K. (2006). Phase I study of EKB-569, an irreversible inhibitor of the epidermal growth factor receptor, in patients with advanced solid tumors. J. Clin. Oncol..

[41] Sp N., Kang D.Y., Joung Y.H., Park J.H., Kim W.S., Lee H.K., Song K.D., Park Y.M., Yang Y.M. (2017). Nobiletin Inhibits Angiogenesis by Regulating Src/FAK/STAT3-Mediated Signaling through PXN in ER+ Breast Cancer Cells. Int. J. Mol. Sci..

[42] Kang D.Y., Sp N., Kim D.H., Joung Y.H., Lee H.G., Park Y.M., Yang Y.M. (2018). Salidroside inhibits migration, invasion and angiogenesis of MDAMB 231 TNBC cells by regulating EGFR/Jak2/STAT3 signaling via MMP2. Int. J. Oncol..

[43] Chen J., Jiang C.C., Jin L., Zhang X.D. (2016). Regulation of PD-L1: A novel role of pro-survival signalling in cancer. Ann. Oncol..

[44] Abdelhamed S., Ogura K., Yokoyama S., Saiki I., Hayakawa Y. (2016). AKT-STAT3 Pathway as a Downstream Target of EGFR Signaling to Regulate PD-L1 Expression on NSCLC cells. J. Cancer.

[45] Ogawara Y., Kishishita S., Obata T., Isazawa Y., Suzuki T., Tanaka K., Masuyama N., Gotoh Y. (2002). Akt enhances Mdm2-mediated ubiquitination and degradation of p53. J. Biol. Chem..

[46] Gong A.Y., Zhou R., Hu G., Li X., Splinter P.L., O’Hara S.P., LaRusso N.F., Soukup G.A., Dong H., Chen X.M. (2009). MicroRNA-513 regulates B7-H1 translation and is involved in IFN-gamma-induced B7-H1 expression in cholangiocytes. J. Immunol..

[47] Guo W., Tan W., Liu S., Huang X., Lin J., Liang R., Su L., Su Q., Wang C. (2015). MiR-570 inhibited the cell proliferation and invasion through directly targeting B7-H1 in hepatocellular carcinoma. Tumour Biol..

[48] Lowe S.W., Bodis S., Mcclatchey A., Remington L., Ruley H.E., Fisher D.E., Housman D.E., Jacks T. (1994). P53 Status and the Efficacy of Cancer-Therapy in-Vivo. Science.

[49] Santoro A., Vlachou T., Luzi L., Melloni G., Mazzarella L., D’Elia E., Aobuli X., Pasi C.E., Reavie L., Bonetti P. (2019). p53 Loss in Breast Cancer Leads to Myc Activation, Increased Cell Plasticity, and Expression of a Mitotic Signature with Prognostic Value. Cell Rep..

[50] Chiarugi V., Ruggiero M. (1996). Role of three cancer “master genes” p53, bcl2 and c-myc on the apoptotic process. Tumori.

[51] Espinosa E., Zamora P., Feliu J., Baron M.G. (2003). Classification of anticancer drugs - a new system based on therapeutic targets. Cancer Treat. Rev..

[52] Sun J.C., Wei Q., Zhou Y.B., Wang J.Q., Liu Q., Xu H. (2017). A systematic analysis of FDA-approved anticancer drugs. BMC Syst. Biol..

[53] Xu X.L., Gu H., Wang Y., Wang J., Qin P. (2019). Autoencoder Based Feature Selection Method for Classification of Anticancer Drug Response. Front Genet..

[54] Schiller J.H., Harrington D., Belani C.P., Langer C., Sandler A., Krook J., Zhu J.M., Johnson D.H., Grp E.C.O. (2002). Comparison of four chemotherapy regimens for advanced non-small-cell lung cancer. New Engl. J. Med..

[55] Hanna N., Shepherd F.A., Fossella F.V., Pereira J.R., De Marinis F., von Pawel J., Gatzemeier U., Tsao T.C.Y., Pless M., Muller T. (2004). Randomized phase III trial of pemetrexed versus docetaxel in patients with non-small-cell lung cancer previously treated with chemotherapy. J. Clin. Oncol..

[56] Winton T., Livingston R., Johnson D., Rigas J., Johnston M., Butts C., Cormier Y., Goss G., Inculet R., Vallieres E. (2005). Vinorelbine plus cisplatin vs. observation in resected non-small-cell lung cancer. New Engl. J. Med..

[57] Le Chevalier T., Arriagada R., Le Pechoux C., Grunenwald D., Dunant A., Pignon J.P., Tarayre M., Abratt R., Arriagada R., Bergman B. (2004). Cisplatin-based adjuvant chemotherapy in patients with completely resected non-small-cell lung cancer. New Engl. J. Med..

[58] Sp N., Kang D.Y., Kim D.H., Park J.H., Lee H.G., Kim H.J., Darvin P., Park Y.M., Yang Y.M. (2018). Nobiletin Inhibits CD36-Dependent Tumor Angiogenesis, Migration, Invasion, and Sphere Formation Through the Cd36/Stat3/Nf-Kb Signaling Axis. Nutrients.

[59] Kang D.Y., Darvin P., Yoo Y.B., Joung Y.H., Sp N., Byun H.J., Yang Y.M. (2016). Methylsulfonylmethane inhibits HER2 expression through STAT5b in breast cancer cells. Int. J. Oncol..

[60] Sp N., Darvin P., Yoo Y.B., Joung Y.H., Kang D.Y., Kim D.N., Hwang T.S., Kim S.Y., Kim W.S., Lee H.K. (2015). The combination of methylsulfonylmethane and tamoxifen inhibits the Jak2/STAT5b pathway and synergistically inhibits tumor growth and metastasis in ER-positive breast cancer xenografts. BMC Cancer.

[61] Schlessinger J. (2014). Receptor Tyrosine Kinases: Legacy of the First Two Decades. Cold Spring Harbor Perspect. Biol..

[62] Hynes N.E., Lane H.A. (2005). ERBB receptors and cancer: The complexity of targeted inhibitors. Nat. Rev. Cancer.

[63] Yarden Y., Sliwkowski M.X. (2001). Untangling the ErbB signalling network. Nat. Rev. Mol. Cell Biol..

[64] Sforza V., Martinelli E., Ciardiello F., Gambardella V., Napolitano S., Martini G., Della Corte C., Cardone C., Ferrara M.L., Reginelli A. (2016). Mechanisms of resistance to anti-epidermal growth factor receptor inhibitors in metastatic colorectal cancer. World J. Gastroenterol..

[65] Li M., Yang J.Y., Zhou W.L., Ren Y., Wang X.X., Chen H.P., Zhang J.Y., Chen J.L., Sun Y.H., Cui L.J. (2017). Activation of an AKT/FOXM1/STMN1 pathway drives resistance to tyrosine kinase inhibitors in lung cancer. Brit. J. Cancer.

[66] Jacobsen K., Bertran-Alamillo J., Molina M.A., Teixido C., Karachaliou N., Pedersen M.H., Castellvi J., Garzon M., Codony-Servat C., Codony-Servat J. (2017). Convergent Akt activation drives acquired EGFR inhibitor resistance in lung cancer. Nat. Commun..

[67] Sionov R.V., Haupt Y. (1999). The cellular response to p53: The decision between life and death. Oncogene.

[68] Burns T.F., El-Deiry W.S. (1999). The p53 pathway and apoptosis. J. Cell Physiol..

[69] Matsuda K., Tanikawa C. (2017). The transcriptional landscape of p53 signaling pathway. Cancer Res..

[70] Blandino G., Di Agostino S. (2018). New therapeutic strategies to treat human cancers expressing mutant p53 proteins. J. Exp. Clin. Cancer Res..

[71] Zheng L., Ren J.Q., Ll H., Kong Z.L., Zhu H.G. (2004). Downregulation of wild-type p53 protein by HER-2/neu mediated PI3K pathway activation in human breast cancer cells: Its effect on cell proliferation and implication for therapy. Cell Res..

[72] Zhang Y.Z., Han C.Y., Duan F.G., Fan X.X., Yao X.J., Parks R.J., Tang Y.J., Wang M.F., Liu L., Tsang B.K. (2019). p53 sensitizes chemoresistant non-small cell lung cancer via elevation of reactive oxygen species and suppression of EGFR/PI3K/AKT signaling. Cancer Cell Int..

[73] Porta C., Hadj-Slimane R., Nejmeddine M., Pampin M., Tovey M.G., Espert L., Alvarez S., Chelbi-Alix M.K. (2005). Interferons alpha and gamma induce p53-dependent and p53-independent apoptosis, respectively. Oncogene.

[74] Takaoka A., Hayakawa S., Yanai H., Stoiber D., Negishi H., Kikuchi H., Sasaki S., Imai K., Shibue T., Honda K. (2003). Integration of interferon-alpha/beta signalling to p53 responses in tumour suppression and antiviral defence. Nature.

[75] Xue W., Zender L., Miething C., Dickins R.A., Hernando E., Krizhanovsky V., Cordon-Cardo C., Lowe S.W. (2007). Senescence and tumour clearance is triggered by p53 restoration in murine liver carcinomas. Nature.

[76] Menendez D., Shatz M., Azzam K., Garantziotis S., Fessler M.B., Resnick M.A. (2011). The Toll-Like Receptor Gene Family Is Integrated into Human DNA Damage and p53 Networks. PLos Genet..

[77] Shatz M., Menendez D., Resnick M.A. (2012). The Human TLR Innate Immune Gene Family Is Differentially Influenced by DNA Stress and p53 Status in Cancer Cells. Cancer Res..

[78] Textor S., Fiegler N., Arnold A., Porgador A., Hofmann T.G., Cerwenka A. (2011). Human NK Cells Are Alerted to Induction of p53 in Cancer Cells by Upregulation of the NKG2D Ligands ULBP1 and ULBP2. Cancer Res..

[79] Quan X.W., Li X.L., Yin Z.H., Ren Y.W., Zhou B.S. (2019). p53/miR-30a-5p/SOX4 feedback loop mediates cellular proliferation, apoptosis, and migration of non-small-cell lung cancer. J. Cell Physiol..

[80] Biamonte F., Battaglia A.M., Zolea F., Oliveira D.M., Aversa I., Santamaria G., Giovannone E.D., Rocco G., Viglietto G., Costanzo F. (2018). Ferritin heavy subunit enhances apoptosis of non-small cell lung cancer cells through modulation of miR-125b/p53 axis. Cell Death Dis..

[81] Wu Y.L., Chen W.Y., Xu Z.P., Gu W.Y. (2019). PD-L1 Distribution and Perspective for Cancer Immunotherapy-Blockade, Knockdown, or Inhibition. Front. Immunol..

[82] Pardoll D.M. (2012). The blockade of immune checkpoints in cancer immunotherapy. Nat. Rev. Cancer.

