# Supplementary figures and images for: The Inhibitory Mechanisms of Tumor PD-L1 Expression by Natural Bioactive Gallic Acid in Non-Small-Cell Lung Cancer (NSCLC) Cells
Source: Cancers (Basel). 2020 Mar 19;12(3):727. doi: 10.3390/cancers12030727 (PMC7140102; doi:10.3390/cancers12030727)

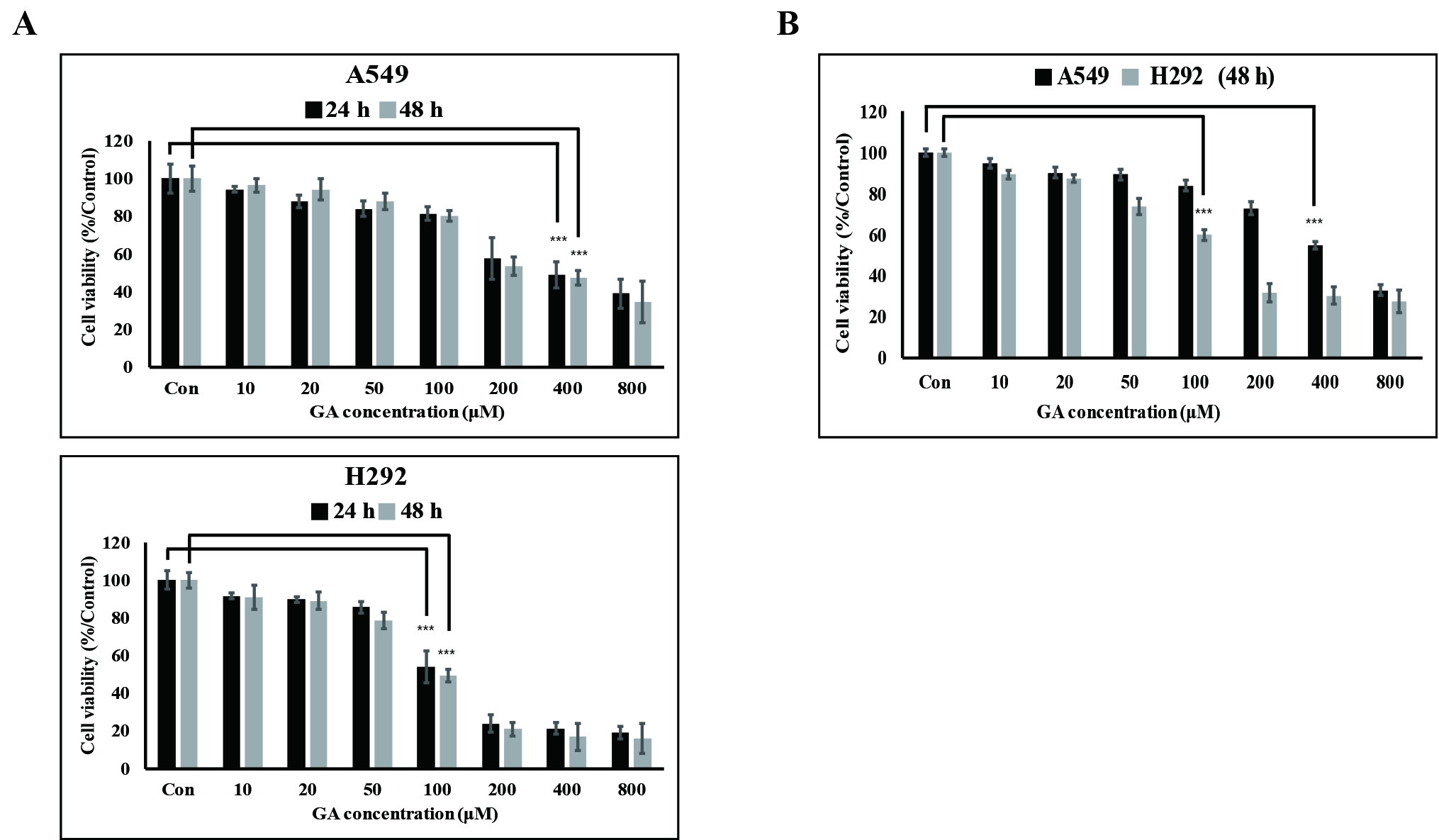

Supplement: Supplementary file 1 [file cancers-12-00727-s001.zip › Supplementary Files/Figure S1.tif]

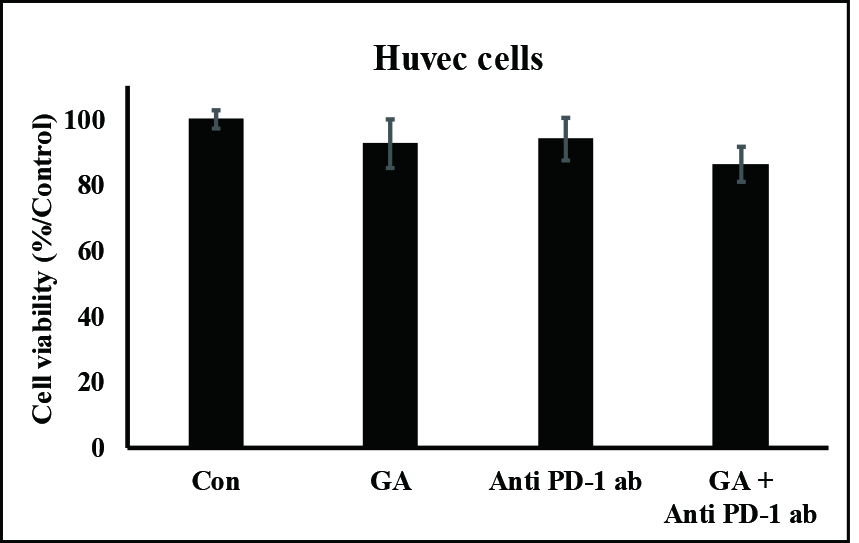

Supplement: Supplementary file 1 [file cancers-12-00727-s001.zip › Supplementary Files/Figure S2.tif]

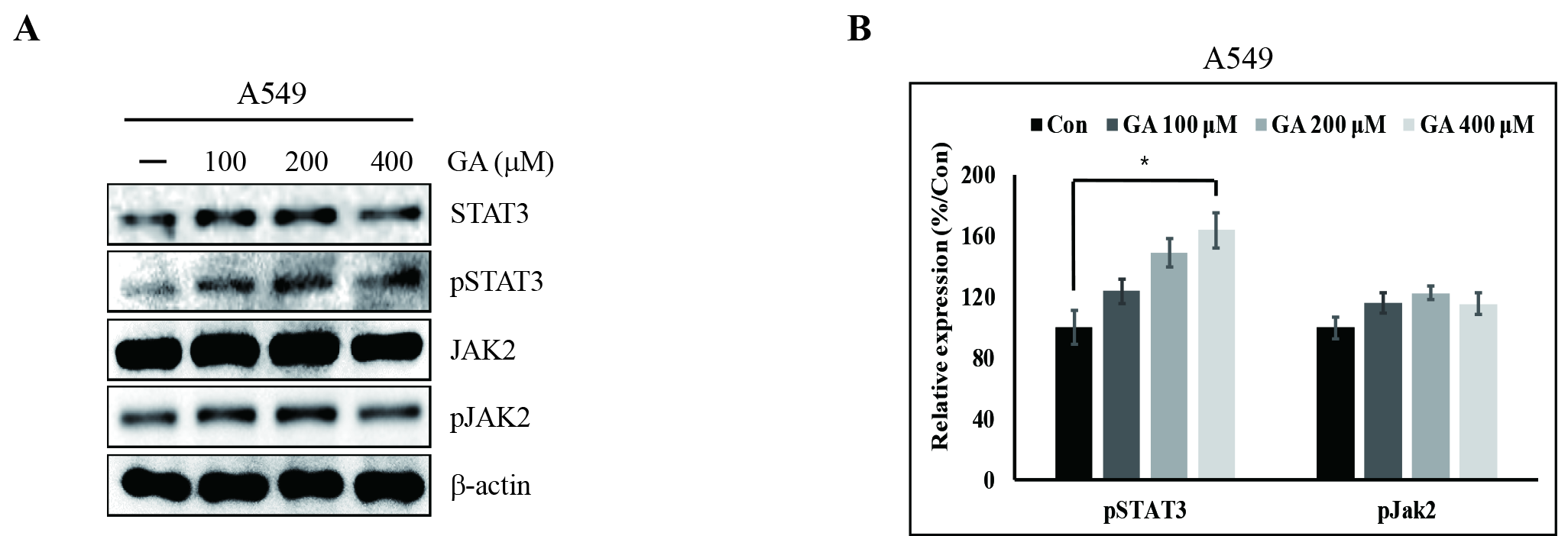

Supplement: Supplementary file 1 [file cancers-12-00727-s001.zip › Supplementary Files/Figure S3.tif]
